# Nutritional Value and Biofunctionalities of Two Edible Green Seaweeds (*Ulva lactuca* and *Caulerpa racemosa*) from Indonesia by Subcritical Water Hydrolysis

**DOI:** 10.3390/md19100578

**Published:** 2021-10-15

**Authors:** Ratih Pangestuti, Monjurul Haq, Puji Rahmadi, Byung-Soo Chun

**Affiliations:** 1 Research & Development Divisions for Marine Bio Industry (BBIL), National Research and Innovation Agency (BRIN), North Lombok 83352, Indonesia; 2 Department of Fisheries & Marine Bioscience, Jashore University of Science & Technology, Jashore 7408, Bangladesh; mr.haq@just.edu.bd; 3 Research Center for Oceanography, National Research and Innovation Agency (BRIN), Jakarta 14430, Indonesia; puji.rahmadi@lipi.go.id; 4 Department of Food Science & Technology, Pukyong National University, 45 Yongso-ro, Nam-gu, Busan 48513, Korea

**Keywords:** *Caulerpa racemosa*, *Ulva lactuca*, nutritional, potential, SWE

## Abstract

*Caulerpa racemosa* (sea grapes) and *Ulva lactuca* (sea lettuces) are edible green seaweeds and good sources of bioactive compounds for future foods, nutraceuticals and cosmeceutical industries. In the present study, we determined nutritional values and investigated the recovery of bioactive compounds from *C. racemosa* and *U. lactuca* using hot water extraction (HWE) and subcritical water extraction (SWE) at different extraction temperatures (110 to 230 °C). Besides significantly higher extraction yield, SWE processes also give higher protein, sugar, total phenolic (TPC), saponin (TSC), flavonoid contents (TFC) and antioxidant activities as compared to the conventional HWE process. When SWE process was applied, the highest TPC, TSC and TFC values were obtained from *U. lactuca* hydrolyzed at reaction temperature 230 °C with the value of 39.82 ± 0.32 GAE mg/g, 13.22 ± 0.33 DE mg/g and 6.5 ± 0.47 QE mg/g, respectively. In addition, it also showed the highest antioxidant activity with values of 5.45 ± 0.11 ascorbic acid equivalents (AAE) mg/g and 8.03 ± 0.06 trolox equivalents (TE) mg/g for ABTS and total antioxidant, respectively. The highest phenolic acids in *U. lactuca* were gallic acid and vanillic acid. Cytotoxic assays demonstrated that *C. racemosa* and *U. lactuca* hydrolysates obtained by HWE and SWE did not show any toxic effect on RAW 264.7 cells at tested concentrations after 24 h and 48 h of treatment (*p* < 0.05), suggesting that both hydrolysates were safe and non-toxic for application in foods, cosmeceuticals and nutraceuticals products. In addition, the results of this study demonstrated the potential of SWE for the production of high-quality seaweed hydrolysates. Collectively, this study shows the potential of under-exploited tropical green seaweed resources as potential antioxidants in nutraceutical and cosmeceutical products.

## 1. Introduction

Seaweed, also known as marine macroalgae, comprises photosynthetic organisms and includes more than 12,000 species [1,2]. Based on photosynthetic pigment, they can be categorized into: Rhodophyceae (red seaweeds), Phaeophyceae (brown seaweeds) and Chlorophyceae (green seaweeds) [3]. Seaweeds play an important ecological, socio-economic role for coastal communities and are also used for many purposes such as food, medicinal, building materials, feed and many others. Seaweeds are rich in bioactive materials such as polysaccharides, proteins, peptides, amino acids and secondary metabolites including polyphenolic compounds and natural pigments. These bioactive materials have been demonstrated to possess various biological activities and medicinal and health beneficial effects. In addition, many studies have found that countries where seaweeds are consumed on a daily basis have significantly fewer diet-related diseases and longer life expectancy [1]. Seaweeds’ bioactive compounds have become a driving factor for their increased demand in food, nutraceutical and cosmeceutical products [4].

Seaweeds are widely distributed and can be found in all zones on the Earth from polar, temperate to tropical regions. Indonesia is an archipelagic country with a long coastline and lies within the heart of the Coral Triangle, the center of the highest marine biodiversity on Earth [5]. The earliest documentation of seaweeds diversity in Indonesia is reported by Rumphius (1750), who established the botanical foundations of the flora of Indonesia. Further, in 1912, van Bosse documented 782 seaweed species in Indonesia, which consisted of 196 species of green seaweeds, 452 species of red seaweeds and 134 species of brown seaweeds. Recently, it was reported that around 1000 seaweeds species can be found in Indonesia [4,6]. Despite the great diversity of seaweed species in Indonesia, only a few species have been used for foods, supplements, nutraceutical and cosmeceutical industries. Among tropical seaweeds species, *Caulerpa racemosa* (known as sea grapes or green caviar) and *Ulva lactuca* (known as sea lettuces) belong to the green algae (Chlorophyta) represent under-exploited seaweed resources in Indonesia. Sulfated polysaccharides from *Ulva* spp. have beneficial effects for cancer chemoprevention, anti-hypertensive and immune-modulating activities [7,8,9]. In addition, the aqueous extract of *Caulerpa* spp. showed anti-photoaging activity in UVB-irradiated mice [10]. Unfortunately, bioactive compounds, as well as biofunctionalities of *C. racemosa* and *U. lactuca* from Indonesia, are not well characterized. In addition, fewer studies were conducted concerning the bioactive compounds from green seaweeds and their biological activities compared to other seaweed classes [11]. 

Generally, hot water extraction (HWE), organic solvents’ extraction and acid/base extraction were used to extract bioactive compounds from seaweeds [4]. However, exposure to organic solvents and strong acids/bases can lead to deleterious effects on human health and environmental concerns. Therefore, environmentally friendly technologies such as microwave-assisted extraction (MAE), ultrasound-assisted extraction (UAE), enzymatic hydrolysis (EAE), ultrasound-assisted extraction (UAE) and subcritical water hydrolysis (SWE) are gaining more attention for development in many sectors. Bioactive compounds such as polysaccharides, carotenoids and phenolic compounds have been extracted from seaweeds by SWE [12,13]. During the SWE process, solvents were maintained in a subcritical state, between boiling point (100 °C; 0.10 MPa) and critical point (374 °C; 22 MPa), where they remain as a liquid due to the high pressure [4]. Temperature is one of the crucial factors that affect the efficiency and selectivity in the SWE process [5]. Previous studies have demonstrated that the seaweed hydrolysates obtained from SWE have better biological activities as compared to hydrolysates obtained by the conventional HWE process [14]. Considering its high productivity, effectiveness, extraction time, low cost and environmental friendliness, the SWE process has shown many benefits over conventional HWE and other extraction methods. 

The main objective of this work was to characterize bioactive compounds in two edible under-exploited tropical seaweeds. First, proximate compositions and fatty acid profiles of *U. lactuca* and *C. racemosa* were analyzed. These two green seaweeds species were further hydrolyzed by conventional HWE at 100 °C and SWE at four different temperature conditions (110 °C, 150 °C, 190 °C and 230 °C). The hydrolysates from HWE and SWE were further analyzed for biochemical compositions, including total protein, sugar, phenolic, flavonoid and saponin contents. Biological activities of *C. racemosa* and *U. lactuca* were tested using radical scavenging assays, and cytotoxic potentials were studied to gain insight into the potential toxicity of seaweeds hydrolysates. The data obtained and presented in this research on the chemical composition of two edible seaweeds can provide the foundations for the explorations of under-exploited seaweeds in Indonesia and fill the gaps for future research in the development of functional foods, nutraceuticals and cosmeceuticals from *U. lactuca* and *C. racemosa*.

## 2. Results and Discussion

### 2.1. Proximate and Fatty Acid Composition of C. racemosa and U. lactuca

Carbohydrates were the major component of *C. racemosa* and *U. lactuca*, accounting for 38.62 ± 0.01 and 61.83 ± 0.01% of the proximate content, respectively (Table 1). Both green seaweeds also contained protein (7.60 ± 0.01 and 10.0 ± 0.01%), ash (38.41 ± 1.90 and 17.86 ± 0.87%), lipids (0.71 ± 0.01 and 0.13 ± 0.01%) and moisture contents (14.66 ± 0.43 and 10.18 ± 0.04%). When *C. racemosa* and *U.lactuca* were compared directly, the protein and carbohydrates contents of *U. lactuca* were higher than those of *C. racemosa*. The carbohydrate contents of *U. lactuca* found in this study were slightly higher than reported in other studies [15,16,17]. For example, Rasyid et al. (2017) reported that carbohydrate contents of *U. lactuca* from Pamengpeu, and West Java–Indonesia were 58.1% [15]. The carbohydrates contents in seaweeds are likely to be dependent on geographic location, the season of harvest and algal maturity [18]. Many studies have reported that seaweeds contain high carbohydrates and/or protein but low lipid contents. High carbohydrate contents in *U. lactuca* suggest that these green seaweeds could be an important source of polysaccharides for industrial uses. One of the major sulfated polysaccharides found in the genera *Ulva* spp. is ulvan, which may constitute 8 to 40% of the seaweed biomass [19]. Although the industrial applications based on ulvans are still limited, these sulfated polysaccharides have been demonstrated to possess a broad range of bioactivities such as immunomodulating, antiviral, antioxidant, antihyperlipidemic and anticancer activities [20]. Ulvan has been demonstrated to promote gastrointestinal health and has been linked to a reduction in the incidence of non-communicable diseases (NCD) [21,22]. Ulvan has the potential to be applied as bioactive compounds in foods, nutraceuticals and cosmeceuticals; however, the structural and biological properties of ulvan from *U. lactuca* require thorough investigation.

In terms of lipid content, the values found in both species were relatively low, indicating that both *C. racemosa* and *U. lactuca* are an ideal choice for people who require a low-fat diet [23]. The differences in proximate contents of seaweeds could be attributed to the differences in species, biological conditions, postharvest treatment and preparative methods. The fatty acids composition (area%) of *C. racemosa* and *U. lactuca* are given in Table 2. The Table 2 shows that 11 of 37 types of authentic standard fatty acids were identified in *U. lactuca,* while 24 of 37 types of authentic standard fatty acids were identified in *C. racemosa*. In *C. racemosa* and *U. lactuca*, palmitic acid (C16:0) showed maximum quantities of 50.73 ± 1.41 and 46.64 ± 1.12%, respectively. The proportion of PUFAs in *C. racemosa* found in this study was higher compared to *U. lactuca,* with linolenic acid (C18:3) found as the major omega-3 PUFAs in both species. Our results show that EPA (C20:5n3), DHA (C22:6n3) and AA(C20:4n6) were not detected in both seaweeds, but they contained essential fatty acids such as linoleic acid (C18:2n6) and linolenic acid (C18:3). In contrast, previous studies reported the presence of EPA and DHA in *C. racemosa* and *U. lactuca* [17,24]. It has been reported by Nelson et al. (2002) that variations in fatty acid compositions are attributable to environmental and genetic differences. Ratios of omega-6/omega-3 fatty acids found in this study were relatively low, at 2.71 and 1.18 for *C. racemosa* and *U. lactuca*, respectively. As regards international organizations, the World Health Organization recommends that the ratio of omega-6/omega-3 fatty acids should not exceed 10 in the daily diet [17], since high omega-6/omega-3 fatty acids ratios will increase risk of many diseases [25]. Hence, the low omega-6/omega-3 fatty acids ratios found in *C. racemosa* and *U. lactuca* suggest that both seaweeds are a good source of omega-3 fatty acids and also an important source of supply of omega-3 fatty acids for homeostasis and maintaining human health [26]. 

### 2.2. Extraction Yield of C. racemosa and U. lactuca

**Figure 1:**
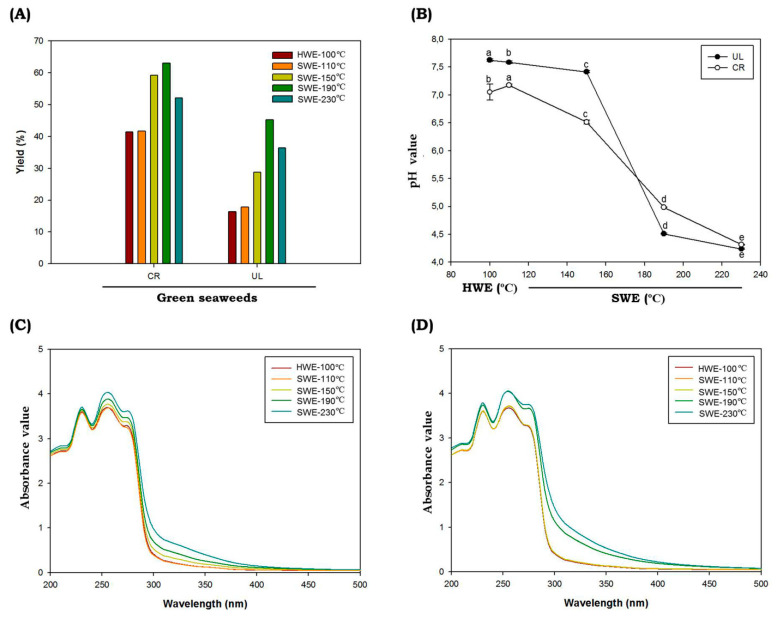
Chemical characteristics of *C. racemosa* and *U. lactuca*. Yield (**A**), pH (**B**), UV-absorbance spectra of *C. racemosa* (**C**) and *U. lactuca* (**D**) obtained by subcritical water hydrolysis. Abbreviations: HWE: hot water extractions; SWE: subcritical water extractions; UL: *Ulva lactuca*; CR: *caulerpa racemosa*. Different letters (a–e) denote a statistically significant difference (*p* < 0.05).

During the hydrolysis process, HWE was maintained at 100 °C and SWE at temperatures of 110 °C, 150 °C, 190 °C and 230 °C. The reaction times taken were 2 h and 10 min for HWE and SWE, respectively. The pressure of the SWE process was monitored using the pressure gauge and maintained at 5–7 MPa. The extraction yields ranged from 16.37 to 36.38% and 41.49 to 52.08% (dry weight) for *C. racemosa and U. lactuca,* respectively (Figure 1A). Compared to the conventional HWE, the SWE process showed higher extraction yields. The temperature and SWE process directly affected the extraction yield of green seaweed and reached the highest yield at 190 °C. It has been reported that temperature is one of the important parameters during the SWE process. The increased extraction yield in SWE at higher temperatures can be correlated with the change in the dielectric constant of water. As the temperatures during SWE process increase, the dielectric constant will also increase; hence, bioactive materials would also increase significantly. Higher temperatures in SWE led to increases in mass transfer, rapid extraction, lower surface tension and higher solubility of bioactive materials [27]. However, some compounds will also be degraded at elevated temperatures. The change in pH value can be related to those processes (Figure 1B). The solvent pH prior to hydrolysis was 7.2, and following the SWE process, seaweed hydrolysate tended to be acidic. The pH reached the lowest value at hydrolysis temperatures of 230 °C, with the value of 4.32 ± 0.01 and 4.24 ± 0.01 for *C. racemosa and U. lactuca,* respectively. The low pH value might correlate with the degradation of sugar into organic acids, which further increased the acidity of green seaweed hydrolysates. In accordance with the findings of our study, Park et al. (2019) found that the pH value of red seaweeds *Porphyra yezoensis* hydrolysates following SWE process ware decreased from 7.15 ± 0.01 to 4.16 ± 0.06 at hydrolysis temperatures of 210 °C [28]. 

The UV absorption spectra of *C. racemosa and U. lactuca* hydrolysates are shown in Figure 1C,D. A peak observed at 235 nm was attributed to n–π* transition; the absorption peak near 275 nm was attributed to n→σ* transition for the amino groups; and the spectral absorption at 300 nm was assigned to n→π* transition for the carbonyl or carboxyl groups [29]. When *C. racemosa and U. lactuca* were hydrolyzed by SWE at temperatures of 190 °C and 230 °C, the intensities of the absorption peaks significantly increased, probably because the total protein and other bioactive compounds such as polyphenolic compounds were higher compared to HWE and SWE at lower temperatures. In addition, *C. racemosa* and *U. lactuca* showed strong absorption in the ultraviolet (UV)-B region around 280 to 320 nm (Figure 1C,D) indicating that both green species were rich in UVB-absorbing compounds. In marine environments, light variations occur on much shorter timescales, ranging from seconds to minutes, hours and even days. As a result, seaweeds including *C. racemosa* and *U. lactuca* must avoid the contradiction between effective light absorption on the one hand and a quick photoprotective response to photoinhibitory light intensities on the other [30]. In addition, Wiraguna et al. (2018) has reported UVB-protective activity of *Caulerpa* sp from Indonesia [31]. The presence of UVB-absorbing compounds in *C. racemosa* and *U. lactuca* will allow for future perspectives to understand the photoprotective mechanisms in these tropical green seaweeds. Further, these UVB-absorbing compounds can be used as UVB filters to absorb the entire spectrum of UVB radiation, and these potential compounds can be delivered for the development in the cosmeceutical applications [4].

Analysis of the seaweed hydrolysates’ color is shown in Table 3, in which the HWE and SWE at low extraction temperature gave the highest lightness (L*) value. One possible reason for the lighter color observed in HWE and SWE at 110 °C is a shorter exposure to the heat treatment as compared to the higher reaction temperatures. The L* value then decreased when the reaction temperature increased [32]. The L* values of hydrolysates obtained in this study ranged from 32.43 to 54.47 and 23.64 to 56.18 for *C. racemosa* and *U. lactuca*, respectively. It can be seen that L* values were remarkably lower in hydrolysates obtained by SWE at higher temperatures (*p* < 0.05), which showed the significant effect of the temperature and hydrolysis process on L* values. Accordingly, redness (a*) and blueness (b*) values were higher as the temperature of SWE increased, and the lowest values were obtained from the HWE process. There was a significant difference due to the hydrolysis process (*p* < 0.05). The chroma (C*) value indicates the degree of saturation of color and is proportional to the strength of the color. In this study, we found changes in variations in C* values between HWE and SWE. In addition, the C* values also varied at different hydrolysis temperatures (*p* < 0.05). The highest C value was found for seaweed hydrolysates obtained by HWE. In addition, hue angle (H*) is another parameter often used to determine the color of hydrolysates. In our study, we found that H* values of seaweed hydrolysates obtained by SWE at higher temperatures (190 °C and 230 °C) were higher than those of HWE and SWE at lower temperatures (*p* < 0.05). Our results showed that the hydrolysis process especially by SWE at higher temperatures gives greater a*, b*, C* and H* to the seaweed hydrolysates. Pourali et al. (2010) reported that dark color following the SWE process might be correlated with the formation of 5-hydroxymethyl-2-furfural (HMF) and soluble polymers from the decomposition of the produced soluble sugars in a subcritical medium [33]. In addition, the dark color observed at higher temperatures is also attributed to the formation of undesired materials undergoing the Maillard reaction products (MRPs). The UV absorbance at 420 nm is often used to monitor the browning intensity caused by brown polymeric substances, such as melanoidins, which are formed at the final phase of MRPs [34]. Temperature is an important parameter of MRPs, as increasing the temperature could reduce the surface tension and viscosity of water, which resulted in an enhanced solubility of the analytes in the solvent, which further increased reaction rate. As demonstrated in Table 4, compared to the HWE, the MRPs levels were increased under the SWE process. The MRPs product level was the highest under SWE extraction conditions of 230 °C (*p* < 0.05). This subset of MRPs contributes to the coloration of many processed products. The intensity of brown color of these extracts increased with elevation in temperature, supporting the occurrence of MRPs during the SWE process. The MRPs provide a unique aroma and changes in food quality parameters. The process could be indicated from the appearance of the extract, as the color of extracts turned dark brown at temperatures above 150 °C. Interestingly, in our study, we noticed a burning odor in the seaweed hydrolysates obtained by SWE at an extraction temperature above 150 °C. Similar observations (in terms of solution color and odor) have been reported in several studies [34,35].

### 2.3. Total Protein, Sugars and Reducing Sugar Contents of C. racemosa and U. lactuca Hydrolysates

**Figure 2:**
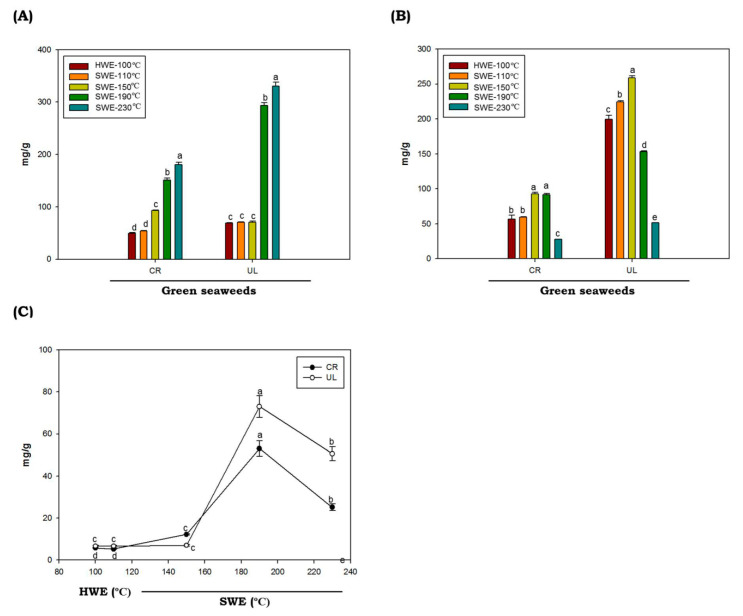
Total protein (**A**), sugar (**B**) and reducing sugar (**C**) of green seaweed hydrolysates obtained by HWE and SWE. CR: *C. racemosa*; UL: *U. lactuca*. Values correspond to mean ± SD from three independent experiments. Different letters (a–e) denote a statistically significant difference (*p* < 0.05).

The total protein contents in *C. racemosa* and *U. lactuca* hydrolysates obtained by HWE and SWE at various temperatures are provided in Figure 2A. In this study, we found that the protein contents of green seaweeds were not significantly different in HWE and SWE processes at extraction temperatures of up to 150 °C (*p* < 0.05). Interestingly, at higher temperatures (above 150 °C), the protein contents were increased dramatically. The highest protein yield (330.37 mg/g ± 5.46) was obtained from the *U. lactuca* hydrolyzed at 230 °C. Protein has low solubility at low temperature, due to robust aggregation via hydrophobic interactions [36,37]. When the temperature rises, the water ionization constant rises, increasing the protein yield observed in *C. racemosa* and *U. lactuca.*

The total sugar values of *C. racemosa* and *U. lactuca* hydrolysates are shown in Figure 2B. The proportions of total sugars of *C. racemosa* and *U. lactuca* ranged from 56.88 to 93.04 mg/g and 51.67 to 258.95 mg/g, respectively. Total sugar of *C. racemosa* and *U. lactuca* with the highest content was obtained by SWE at 150 °C, with values of 93.04 ± 2.13 mg/g and 258.93 ± 2.71 mg/g, respectively. Compared to *C. racemosa, U. lactuca* showed higher total sugar contents. The sugar contents of *U. lactuca* found in this study are comparable to the total sugar contents of *U. lactuca* from Tunisia and Israel, which were 272 mg/g and 68.10 to 159.29 mg/g, respectively [38,39]. However, compared to Arctic *U. lactuca*, the sugar contents found in this study were slightly lower [40]. It was reported that the variations in total sugar contents could be affected by temporal or spatial variations in sugar contents of particular seaweed species, and also by methodological differences. In addition, we found that the levels of total sugars in both green seaweeds were decreased at temperatures above 150 °C. The hydrolysis of poly- or oligosaccharides and the degradation of monosaccharides caused by the high ionic product of solvent at elevated temperature under SWE conditions were thought to be the cause of the decrease in total sugar content [41]. Both *C. racemosa* and *U. lactuca* produce low amounts of reduced sugars when hydrolyzed by HWE or SWE at temperatures of up to 150 °C. The highest reducing sugar levels of both *C. racemosa* and *U. lactuca were* obtained by SWE at temperatures of 190 °C with values of 53.06 ± 3.65 mg/g 73.00 ± 5.15 mg/g, respectively. It was demonstrated that reducing sugar content from the seaweeds polysaccharides by SWE increased up to certain reaction temperatures and then decreased [42]. The lower levels of reducing sugar may be correlated to the decomposition of sugar into other products, such as ketones and aldehydes, from which organic acids can be produced. 

### 2.4. Phenolics, Saponins and Flavonoid Contents of C. racemosa and U. lactuca Hydrolysates

**Figure 3:**
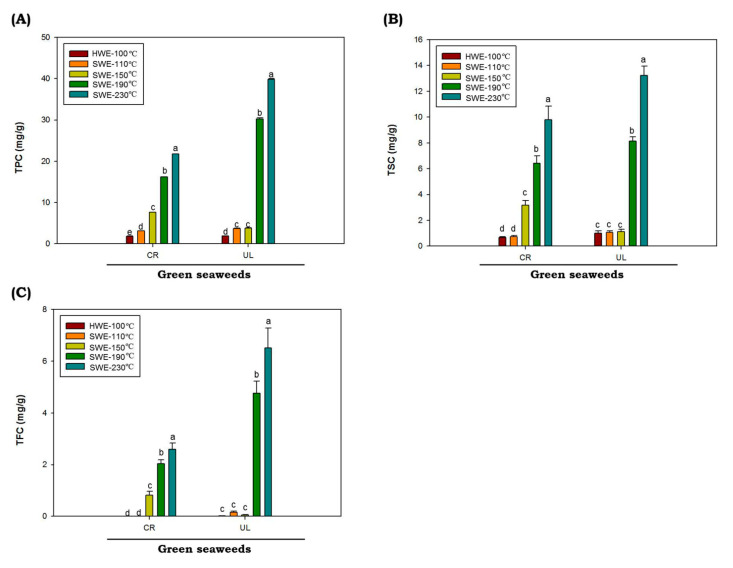
Total phenolic (**A**), flavonoid (**B**) and saponin (**C**) contents of green seaweed hydrolysates were obtained by HWE and SWE. CR: *C. racemosa*; UL: *U. lactuca*. Values correspond to mean ± SD from three independent experiments Different letters (a–e) denote a statistically significant difference (*p* < 0.05).

Polyphenols are naturally present in plants such as seaweeds, which help them to eliminate free radicals. In this study, results for total phenolic (TPC), saponin (TSC) and flavonoid (TFC) *C. racemosa* and *U. lactuca* hydrolysates are shown in Figure 3. The values of TPC, TSC and TFC were represented as gallic acid equivalent (GAE), diosgenin equivalent (DE) and quercetin equivalent (QE), respectively. The values of TPC, TSC and TFC of *C. racemosa* and *U. lactuca* hydrolysates extracted by HWE and SWE at 110 up to 150 °C are low. However, as temperatures increased from 190 to 230 °C, the TPC, TSC and TFC of both *C. racemosa* and *U. lactuca* were significantly increased (*p* < 0.05). The highest TPC, TSC and TFC of both green seaweeds were obtained at reaction temperatures of 230 °C. The TPC, TSC and TFC values were obtained from *U. lactuca* hydrolyzed at 230 °C with the value of 39.82 ± 0.32 GAE mg/g, 13.22 ± 0.33 DE mg/g and 6.5 ± 0.47 QE mg/g, respectively. It has been reported that temperature is one of the most important factors affecting TPC, TSC and TSC in SWE process. In addition, it was reported that when the dielectric constant SWE decreases as the temperature rises, more nonpolar phenolics are being extracted [43].

Total phenolic contents of *C. racemosa* and *U. lactuca* hydrolysates are higher as compared to the TSC and TFC. Therefore, phenolic acid constituents from both green seaweed hydrolysates were quantified by HPLC. The contents of phenolic compounds in *C. racemosa* and *U. lactuca* hydrolysates were estimated based on the reference phenolic acid standards calibration curves. The main constituents of the phenolic acids present in *C. racemosa* and *U. lactuca* are summarized in Table 5. The phenolic acids with the highest levels in *U. lactuca* were gallic acid and vanillic acid. Interestingly, phenolic acids in both green seaweeds hydrolysates obtained by SWE generally increased at elevated temperatures up to 230 °C. However, a previous study reported a loss of phenolic acids, which were hydrolyzed using SWE at high temperatures (above 200 °C). Decreased phenolic acid levels in the SWE hydrolysates at elevated temperatures may be related to the conversion of phenolic acid into decarboxylation products and other gaseous products [44]. At elevated temperatures, phenolic compounds degraded much faster. Khuwijitjaru et al., (2014) demonstrated that only the chlorogenic, p-hydroxybenzoic, protocatechuic and syringic acids were present at 200 °C after 1h of SWE treatments. Notably, in this study, we found an increment in chlorogenic, p-hydroxybenzoic and protocatechuic acid at temperatures of 190 and 230 °C. It was reported that substituent groups on the ring structure of phenolic acids, such as amino, hydroxyl and methoxyl, acted as an activating group in the SWE process, assisting the thermal decarboxylation of benzoic acid derivatives [45]. 

### 2.5. Potential of Cytotoxic and Antioxidant Activities of C. racemosa and U. lactuca Hydrolysates

**Figure 4:**
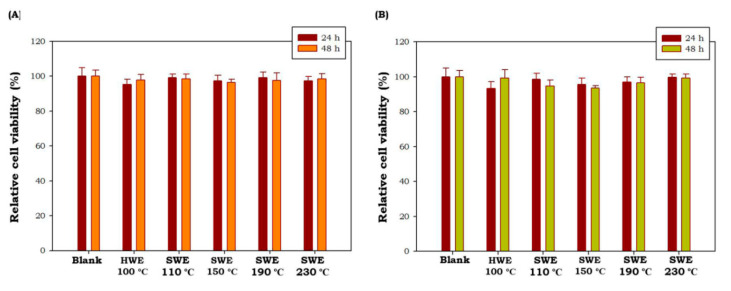
Effects of *C. racemosa* (**A**) and *U. lactuca* (**B**) on the viability of RAW 264.7 cells. HWE: hot water extraction; SWE: subcritical water extraction. Results are the percentage of three independent experiments and are shown as the percentage of viable cells compared with the viability of untreated cells. Values correspond to mean ± SD from three independent experiments.

Potential cytotoxic effects *C. racemosa and U. lactuca* hydrolysates were tested at 50 μg/mL using MTT cell viability assay in cultured macrophage (RAW 264.7 cells). As shown in Figure 4, all green seaweed hydrolysates obtained by HWE as well as SWE did not show any toxic effect on RAW 264.7 cells at tested concentrations after 24 h and 48 h of treatment (*p* < 0.05). These results showed that *C. racemosa* and *U. lactuca* hydrolysates were safe and non-toxic. Similar non-toxic properties of *C. racemosa* and *U. lactuca* aqueous extracts have been reported by previous studies [46,47]. These results showed the potential of *C. racemosa* and *U. lactuca* to be developed in nutraceutical and cosmeceutical products. 

Seaweeds, including green seaweeds, have been continuously demonstrated to possess a wide range of bioactive materials as well as biological activities [10]. In this study, the antioxidant potential of green seaweed hydrolysates obtained by HWE and SWE was tested using ABTS radical scavenging and total antioxidant assays, which are represented as ascorbic acid equivalents (AAE) and trolox equivalents (TE), respectively. The antioxidant potentials of *C. racemosa and U. lactuca* hydrolysates are shown in Table 6. The antioxidant activity of both *C. racemosa* and *U. lactuca* reaches a maximum value with SWE at 230 °C. During the subcritical process at certain temperatures, solvents could extract more bioactive compounds that could not be extracted at lower temperatures and/or by conventional HWE. A temperature of 230 °C was found to be the most optimal condition to obtain bioactive materials from *C. racemosa* and *U. lactuca* using SWE. It was reported that the potential antioxidant activity of *U. lactuca* could be attributed to the higher content of polyphenols, flavonoids, saponins and sulfated polysaccharides compounds, with a known ability to scavenge synthetic radicals in in vitro tests (i.e ABTS) [48]. In addition, in our previous study, we found that the antioxidant activities of seaweeds are strongly correlated with their phenolic contents [5]. The results of the present study demonstrated that green seaweed hydrolysates obtained by SWE could be effective and safe alternatives to fight against radicals. In addition, green seaweed hydrolysates could be used as effective sources for antioxidative nutraceutical and cosmeceutical ingredients. Furthermore, more attention has been raised about the use of natural antioxidants as “natural” entities in nutraceutical and cosmeceutical products [49,50]. These will increase the potency of seaweeds extracts obtained by green extraction methods in various industries since it is of natural origin and environmental friendly. 

## 3. Materials and Methods

### 3.1. Materials

Two under-exploited green seaweed species (*C. racemosa* and *U. lactuca*) were collected from Tual, Southeast Maluku, in June 2018. A voucher specimen was deposited in Balai Bioindustri Laut (BBIL), Lembaga Ilmu Pengetahuan Indonesia (LIPI) West Nusa Tenggara with the accession numbers of GSW-CR-180601 and GSW-UL-180602 for *Caulerpa racemosa* and *Ulva lactuca*, respectively. All the chemicals utilized in this study were obtained from Merck and Junsei Chemical Co., Ltd. (Tokyo, Japan) and were of analytical grade. 

### 3.2. C. racemosa and U. lactuca Sample Preparation

Both *C. racemosa* and *U. lactuca* were washed with clean water; sand debris and other dirt were gently removed. The green seaweeds were further oven-dried at 45 °C for 120 h. In the next step, dried green seaweeds were further freeze-dried and then powderized into a very fine particle (passed through a 0.71 mm siever). The green seaweeds were further kept at −20 °C prior to analysis. 

### 3.3. Proximate Analysis of C. racemosa and U. lactuca

The protein, ash, lipid, protein and moisture contents of *C. racemosa* and *U. lactuca* were measured according to the Association of Official Analytical Chemists methods [51]. Further, total carbohydrate content was estimated by subtracting the total mass of green seaweeds from the sum of other proximate contents.

### 3.4. Fatty Acid Composition Analysis of C. racemosa and U. lactuca

The fatty acid composition of *C. racemosa* and *U. lactuca* were determined using a Fatty Acid Composition Analysis (Agilent Technologies, Wilmington, NC, USA) gas chromatograph with a fused silica capillary column (Supelco, Bellefonte, PA, USA). Methylation of fatty acids (fatty acid methyl esters; FAMEs Supelco, Bellefonte, PA, USA) were prepared according to The American Oil Chemists’ Society’s protocols. The oven temperature was turned on at 130 °C and run for 180 s, and then the temperatures were increased up to 240 °C at a rate of 4 °C/min and then maintained at 240 °C for 600 s. Both the injector and the detector were set to 250 °C. The FAMEs were identified by comparison of retention time with a standard fatty acid methyl ester mixture (Supelco, Bellefonte, PA, USA).

### 3.5. Sample Extraction

#### 3.5.1. Hot Water Extraction of Green Seaweeds

Fine powder of *C. racemosa* and *U. lactuca* was mixed with distilled water at normal pH (7.2) with the sample to solvent ratios of 1:40 (*w/v*). The mixtures were kept at 100 °C and agitated (200 rpm) for 2 h. The hydrolysate obtained after HWE processes was filtered and freeze-dried.

#### 3.5.2. Subcritical Water Extraction (SWE) of Green Seaweeds

The SWE was operated in a continuous-type subcritical water system (Phosentech, South Korea). Fine powder of *C. racemosa* and *U. lactuca* was added into the reactor with distilled water at normal pH (7.2) at 1:40 ratios (*w/v*). The chamber was sealed tightly, purged with nitrogen gas and kept at the desired reaction temperature, pressure and speed (200 rpm) for 10 min. Hydrolyzed green seaweeds were immediately collected after the reaction was terminated and filtered with 0.45 μm membrane filter. The hydrolysate obtained after SWE processes was freeze-dried.

(1) $$\mathrm{Yield}\;\left(\%\right)=\frac{\mathrm{Whyd}}{W0}\times 100\;\%$$

where Whyd is the weight of freeze-dried hydrolysate and W_0_ is the initial weight of green seaweeds.

### 3.6. Physical Properties of C. racemosa and U. lactuca (Color, pH and Maillard Reaction Products (MRPs))

Color properties of *C. racemosa* and *U. lactuca* hydrolysates were measured using a chromameter (Lovibond RT Series, Amesbury (Wiltshire), UK) [5]. The color characteristics of *C. racemosa* and *U. lactuca* were distinguished based on lightness value (L*), redness value (a*) and yellowness value (b*). Chroma meter was standardized each time with black and white references prior to analysis. The hue angle (h*ab) and chroma (C*ab) of *C. racemosa* and *U. lactuca* hydrolysates were calculated based on the following equations:

(2) $$H{}^{\circ}=\tan^{-1}\left(\frac{b*}{a*}\right)$$

(3) $$C*ab=\sqrt{{\left(a*\right)}^{2}+{\left(b*\right)}^{2}}$$

Following the hydrolysis process, *C. racemosa* and *U. lactuca* were filtered and cooled down, and then pH was measured by using a pH meter (Mettler-Toledo, Greifensee, Switzerland). The MRPs were determined through the UV absorbance of samples, as described previously [52]. After the hydrolysis processes, 0.2 mL of filtered *C. racemosa* and *U. lactuca* (1 mg per mL) was measured at 294 and 420 nm.

### 3.7. Total Protein, Total Sugar and Reducing Sugar of C. racemosa and U. lactuca

The protein concentrations of *C. racemosa* and *U. lactuca* hydrolysates were determined following Lowry’s method. The *C. racemosa* and *U. lactuca* hydrolysates (0.2 mL) were mixed with CuSO_4_ reagent at 1:10 ratios (*v/v*) and vortexed. After incubation for 600 s at RT, 0.2 mL of 0.2 N Folin–Ciocalteu reagent (FCR) was loaded into the mixture and incubated for another 0.5 h. Total protein was determined through the UV absorbance of samples at 660 nm, and bovine serum albumin was used as the reference standard.

The total sugar value of *C. racemosa* and *U. lactuca* hydrolysates was measured based on the phenol sulfuric acid method. The *C. racemosa* and *U. lactuca* (0.2 mL) were mixed with 5% phenol (0.2 mL) and sulfuric acid (H_2_SO_4;_ 1 mL) and 0.5 h at 100 °C. The total sugar was determined through the UV absorbance of samples at 490 nm, and C_6_H_12_O_6_ was used as the reference standard.

Reducing sugar analyses of *C. racemosa* and *U. lactuca* hydrolysates were measured by using the 3,5-dinitrosalicylic (DNS) acid method with slight modifications. *C. racemosa* and *U. lactuca* hydrolysates (0.5 mL) were mixed with DNS reagent solution at 1:1 ratios. The mixtures were then incubated at 95 °C for 15 min. After incubation, 0.5 mL of KNaC_4_H_4_O_6_·4H_2_O (40%) was added. Reducing sugar was determined through the UV absorbance of samples at 575 nm, and C_6_H_12_O_6_ was used as the reference standard.

### 3.8. Total Flavonoid Content (TFC), Total Phenolic Content (TPC) and Total Saponin Content (TSC) of C. racemosa and U. lactuca

The TFC of *C. racemosa* and *U. lactuca* hydrolysates were measured according to previous methods [53]. Green seaweed hydrolysates (0.2 mL) were mixed with 0.4 mL of H_2_O and 0.2 mL of 5% NaNO_2_ and incubated at RT for 10 min. Following incubation periods, 10% AlCl_3_ (0.03 mL) and 1 M NaOH (0.4 mL) were added. The mixtures were loaded onto 96-well plates, and the absorbance was measured at 510 nm using multimode microplate readers. Quercetin (Q) was used as the reference standard for flavonoids. The original reaction solution was used to convert the value of the diluted samples. The final results were given in mg Q equivalent/g dry weight (mg Q/g DW).

The TPC of *C. racemosa* and *U. lactuca* hydrolysates was measured using FCR methods [54]. The *C. racemosa* and *U. lactuca* (0.5 mL) were mixed with 0.2 N FCR solution (0.5 mL) and kept in the dark at RT for 10 min. A 7.5% mixture of Na_2_CO_3_ was added (0.5 mL) and kept in the dark at RT for 2 h. The TPC was determined through the UV absorbance of samples at 765 nm, and the final values were expressed as mg phloroglucinol equivalent/g dry weight (mg/g DW).

The TSC of *C. racemosa* and *U. lactuca* hydrolysates was measured using the methods described previously with slight modifications. *C. racemosa* and *U. lactuca* were placed into tubes, at volumes with MeOH at 80% and 0.25 mL; 0.25 mL of 8% vanillin reagent and 2.5 mL H_2_O_4_S (72%) were added. The mixtures were mixed properly and kept at 60 °C for 10 min. After 10 min, the mixtures were transferred into ice. The TSC was determined through the UV absorbance of samples at 544 nm, and the final values were expressed as mg diosgenin equivalent/g dry weight (mg/g DW).

### 3.9. High-Performance Liquid Chromatography (HPLC) Analysis of C. racemosa and U. lactuca Hydrolysates

The *C. racemosa* and *U. lactuca* hydrolysates were further analyzed for phenolic acid compositions using the HPLC system (Hitachi America Ltd., White Plains, NY, USA) on a Nucleosil C_8_ column (Macherey-Nagel, Düren, Germany) with linear gradients of solvent A (H_2_O with 0.1% CH_3_COOH) and solvent B (C_2_H_3_N with 0.1% CH_3_COOH) at a flow rate of 1 mL per min. The elution peaks were detected at 280 nm. The HPLC peak was confirmed with the reference phenolic acids and expressed as mg/g DW.

### 3.10. Antioxidant Activity

#### 3.10.1. 2,2-Azino-bis(3-ethylbenzothiazoline-6-sulfonic acid) Scavenging Assay

The 2,2-azino-bis(3-ethylbenzothiazoline-6-sulfonic acid) (ABTS) (7 mmol/L) and K_2_S_2_O_8_ (2.45 mmol/L) were prepared in a separate bottle, and both solutions were kept in the dark at RT. After 24 h, both solutions were mixed. The radical mixtures were diluted with MeOH to obtain absorbance values of 0.72. The *C. racemosa* and *U. lactuca* hydrolysates were mixed with ABTS radical mixture at 1:5 ratios (*v/v*). The ABTS scavenging activity was determined through the UV absorbance of samples at 734 nm, and MeOH was used as the negative control. In comparison, different concentrations of ascorbic acid were used as standard and evaluated. The results are expressed in terms of AAE.

#### 3.10.2. Total Antioxidant Capacity (TAC)

The *C. racemosa* and *U. lactuca* hydrolysates (0.1 mL) were mixed with 3 mL of radical mixture consist of 0.6 M H_2_SO_4_, 28 mM Na_3_PO_4_ and 4 mM (NH_4_)_6_Mo_7_O_24_. The mixtures were maintained at 95 °C for 180 min. The total antioxidant activity was determined through the measurements of UV absorbance at 695 nm, and MeOH was used as negative control. In comparison, different concentrations of trolox were used as standard and evaluated. The results are expressed in terms of TE.

### 3.11. Effects of Seaweeds Hydrolysates on Cell Viability

Cytotoxic effects of *C. racemosa* and *U. lactuca* hydrolysates were determined by MTT reduction assay [55]. First, macrophage (RAW 264.7) cells were seeded into cell culture plates at a cell density of 2 × 10^4^ cells/well in serum-free DMEM. The *C. racemosa* and *U. lactuca* hydrolysates (50 μg/mL) were then loaded in the cell culture and then incubated for 24 h. One hundred microliters of an MTT (0.5 mg/ml) solution was loaded into the cultures, and incubation was continued for another 240 min. MTT was used as an indicator of cell viability through its mitochondrial reduction to formazan [56]. The absorbance was measured at 540 nm by using a microplate reader. RAW 264.7 cell viability was calculated by comparison of the absorbance of the control group with treated groups.

### 3.12. Statistical Analysis

The data were presented as means ± SD (*n* = 3). Differences between the means of the individual groups were assessed by one-way ANOVA with Duncan’s multiple range tests. Differences were considered significant at *p* < 0.05. The statistical software package, SPSS v.16 (SPSS Inc., Chicago, IL, USA), was used for the analysis.

## 4. Conclusions

Green seaweed hydrolysates, *C. racemosa* and *U. lactuca,* were prepared via HWE and SWE. Compared to HWE, the SWE process showed higher extraction yields, bioactive compounds and antioxidant activities of seaweed hydrolysates. In addition, six phenolic acids, including gallic acids, chlorogenic acid, gentisic acid, procatechuic acid, *p*-hydroxybenzoic acid and vanillic acid were identified in *U. lactuca* hydrolysates obtained by SWE at 230 °C. The SWE-enabled recovery of bioactive compounds from *C. racemosa* and *U. lactuca* with hydrolysis temperature at 230 °C was found to be the most optimum conditions to obtain bioactive materials with good radical scavenging activities, making it a potential candidate for antioxidant compounds. Collectively, this study provides the foundations for exploring under-exploited tropical green seaweeds and filling the gaps for future research in the development of nutraceuticals and cosmeceuticals from sea grape and sea lettuces.

## Figures and Tables

**Table 1:** Proximate composition of seaweeds.

	Moisture	Ash	Lipid	Protein	Carbohydrate
*Caulerpa racemosa*	14.66 ± 0.43	38.41 ± 1.90	0.71 ± 0.01	7.60 ± 0.01	38.62 ± 0.01
*Ulva lactuca*	10.18 ± 0.04	17.86 ± 0.87	0.13 ± 0.01	10.0 ± 0.01	61.83 ± 0.01

**Table 2:** Fatty acid compositions of two green seaweeds from Indonesia.

Fatty Acids	*C. racemosa*	*U. lactuca*
Caproic acid (C6:0)	0.40 ± 0.01	ND
Capric acid (C10:0)	ND	ND
Undecanoic acid (C11:0)	0.62 ± 0.02	ND
Lauric acid (C12:0)	0.61 ± 0.02	ND
Tridecanoic acid (C13:0)	0.51 ± 0.02	ND
Mystric acid (C14:0)	3.87 ± 0.07	4.05 ± 0.42
Myristoleic acid (C14:1n5)	0.73 ± 0.02	ND
Pentadaecanoic acid (C15:0)	0.71 ± 0.02	4.05 ± 0.42
Palmitic acid (C16:0)	50.73 ± 1.41	46.62 ± 1.12
cis-10-pentadecanoic acid (C15:1)	2.48 ± 0.07	1.92 ± 0.03
Palmitoleic acid (C16:1)	3.45 ± 0.09	2.83 ± 0.07
Cis-10-heptadecanoic acid (C17:0)	0.44 ± 0.01	ND
Stearic acid (C18:0)	3.51 ± 0.09	3.31 ± 0.30
Linolelaidic acid (C18:2n6)	2.50 ± 0.28	ND
Eleic acid	3.27 ± 0.09	5.41 ± 0.17
Elaidic acid	0.37 ± 0.01	17.46 ± 0.46
Arachidic acid (C20:0)	ND	ND
Linoleic acid (C18:2n6)	3.69 ± 0.10	2.59 ± 0.19
Linolenic acid (C18:3)	2.59 ± 0.09	2.20 ± 0.08
cis-11,14-eicosadienoic acid (C20:2)	0.47 ± 0.06	ND
cis-8,11,14-eicosatrienoic acid (C20:3n3)	ND	ND
cis-11,14,17-eicosatrienoic acid (C20:3n3)	ND	ND
cis-5,8,11,14,17-eicosapentanoic acid (20:5n3, EPA)	ND	ND
Behenic acid (C22:0)	1.83 ± 0.05	3.40 ± 0.10
Erucic acid (C22:1)	2.30 ± 0.40	ND
cis-13, 16-docosadienoic acid (C22:2)	0.37 ± 0.00	ND
cis-4,7,10-13,16,19-docosahexanoic acid (DHA) (C22:6n3)	ND	ND
Arachidonic acid (C20:4n6)	ND	ND
Tricosanoic acid (C23:0)	0.54 ± 0.01	ND
Lignoceric acid (C24:0)	11.65 ± 0.33	ND
Nervonic acid (C24:1)	2.19 ± 0.06	ND
Σω-3 PUFAs	2.59	2.20
Σω-6 PUFAs	7.03	2.59
ΣPUFAs	9.62	4.79
ΣSFAa	88.07	95.21
ΣMUFAs	2.30	-
Σω-3/ Σω-6	0.37	0.85
Σω-6/ Σω-3	2.71	1.18

Values are means±standard deviations (*n* = 3). Abbreviations: ND: not detected, ω-3: omega-3; ω-6: omega-6; PUFAs: polyunsaturated fatty acids; SFAs: saturated fatty acids; MUFAs: monounsaturated fatty acids.

**Table 3:** Color characteristics of green seaweed hydrolysates.

Green Seaweed	Conditions	L*	a*	b*	C*	H*
*C. racemosa*	HWE	52.57 ± 0.57 ^a^	−0.75 ± 0.01 ^d^	10.15 ± 0.21 ^e^	10.17 ± 0.21 ^e^	−4.20 ± 0.04 ^e^
	SWE 110 °C	54.57 ± 0.35 ^b^	−0.87 ± 0.01 ^d^	14.54 ± 0.14 ^d^	14.57 ± 0.14 ^d^	−3.40 ± 0.01 ^d^
	SWE 150 °C	36.13 ± 0.37 ^d^	11.13 ± 0.11 ^c^	38.91 ± 0.19 ^c^	40.46 ± 0.21 ^c^	15.96 ± 0.07 ^b^
	SWE 190 °C	32.43 ± 0.23 ^e^	17.58 ± 0.01 ^a^	45.60 ± 0.08 ^b^	48.86 ± 0.06 ^b^	21.08 ± 0.03 ^a^
	SWE 230 °C	39.33 ± 0.40 ^c^	14.49 ± 0.13 ^b^	51.52 ± 0.27 ^a^	53.52 ± 0.30 ^a^	15.70 ± 0.06 ^c^
*U. lactuca*	HWE	56.18 ± 0.61 *^a^*	−1.59 ± 0.04 *^e^*	19.84 ± 0.82 *^e^*	19.90 ± 0.82 *^e^*	−4.57 ± 0.09 *^e^*
	SWE 110 °C	55.81 ± 0.26 *^a^*	−1.07 ± 0.00 *^d^*	22.33 ± 0.52 *^c^*	22.35 ± 0.52 *^c^*	−2.74 ± 0.06 *^d^*
	SWE 150 °C	42.65 ± 0.53 *^b^*	0.72 ± 0.03 *^c^*	21.51 ± 0.35 *^d^*	21.52 ± 0.35 *^d^*	1.92 ± 0.04 *^c^*
	SWE 190 °C	25.07 ± 0.42 *^c^*	24.04 ± 0.08 *^b^*	36.73 ± 0.64 *^a^*	43.89 ± 0.58 *^b^*	33.21 ± 0.37 *^b^*
	SWE 230 °C	23.64 ± 0.05 *^d^*	27.58 ± 0.28 *^a^*	35.92 ± 0.34 *^b^*	45.29 ± 0.44 *^a^*	37.52 ± 0.02 *^a^*

Abbreviations: HWE: hot water extraction; SWE: subcritical water extraction; L*: lightness; a*: red/green coordinate; b*: yellow/blue coordinate; C*: chroma; H*: hue. Values correspond to mean ± SD from three independent experiments. Different letters (a–d, *a*–*d*) denote a statistically significant difference (*p* < 0.05).

**Table 4:** Maillard Reaction Products (MRPs) of green seaweed hydrolysates.

Green Seaweed	Conditions	294	420	294/420
*C. racemosa*	HWE	0.84 ± 0.02 ^d^	0.05 ± 0.00 ^d^	17.23 ± 0.48 ^a^
	SWE 110 °C	0.84 ± 0.06 ^d^	0.05 ± 0.00 ^d^	16.41 ± 1.08 ^b^
	SWE 150 °C	0.98 ± 0.01 ^c^	0.07 ± 0.00 ^c^	13.38 ± 0.12 ^c,d^
	SWE 190 °C	1.17 ± 0.01 ^b^	0.09 ± 0.00 ^b^	12.77 ± 0.16 ^d^
	SWE 230 °C	1.49 ± 0.01 ^a^	0.11 ± 0.00 ^a^	14.05 ± 0.27 ^c^
*U. lactuca*	HWE	0.86 ± 0.01 *^d^*	0.05 ± 0.00 *^d^*	16.41 ± 0.34 *^a^*
	SWE 110 °C	0.86 ± 0.02 *^d^*	0.05 ± 0.00 *^d^*	16.43 ± 0.09 *^a^*
	SWE 150 °C	0.88 ± 0.01 *^c^*	0.06 ± 0.00 *^c^*	14.52 ± 0.35 *^b^*
	SWE 190 °C	1.62 ± 0.02 *^b^*	0.13 ± 0.00 *^b^*	12.46 ± 0.14 *^d^*
	SWE 230 °C	1.99 ± 0.01 *^a^*	0.15 ± 0.00 *^a^*	13.09 ± 0.08 *^c^*

Abbreviations: MRPs: Maillard Reaction Products; HWE: hot water extraction; SWE: subcritical water extraction. Values correspond to mean ± SD from three independent experiments. Values correspond to mean ± SD from three independent experiments. Different letters (a–d, *a*–*d*) denote a statistically significant difference (*p* < 0.05).

**Table 5:** Phenolic acid constituents of green seaweed hydrolysates obtained by HWE and SWE (mg/g dry material).

Green Seaweed	Conditions	Gallic Acid	Chlorogenic Acid	Gentisic Acid	Protocatechuic Acid	*p*-Hydroxybenzoic Acid	Vanillic Acid
*C. racemosa*	HWE	9.26 ± 0.06 ^c^	ND	ND	ND	ND	ND
	SWE 110 °C	7.52 ± 0.17 ^d^	ND	ND	ND	ND	ND
	SWE 150 °C	15.74 ± 0.38 ^a^	0.22 ± 0.02 ^c^	ND	ND	ND	ND
	SWE 190 °C	16.11 ± 0.07 ^a^	1.32 ± 0.04 ^b^	14.62 ± 0.32 ^b^	ND	6.95 ± 0.08 ^b^	ND
	SWE 230 °C	13.91 ± 0.11 ^b^	1.41 ± 0.04 ^a^	27.40 ± 0.51 ^a^	ND	11.21 ± 0.21 ^a^	ND
*U. lactuca*	HWE	9.25 ± 0.05 *^e^*	ND	ND	ND	ND	ND
	SWE 110 °C	14.47 ± 0.21 *^d^*	ND	ND	ND	ND	ND
	SWE 150 °C	26.84 ± 0.19 *^b^*	ND	ND	ND	ND	ND
	SWE 190 °C	31.27 ± 0.58 *^a^*	3.83 ± 0.07 *^b^*	11.69 ± 0.28 *^b^*	1.31 ± 0.04 *^b^*	5.03 ± 0.12 *^a^*	47.15 ± 0.56 *^a^*
	SWE 230 °C	19.74 ± 0.44 *^c^*	5.39 ± 0.15 *^a^*	20.63 ± 0.45 *^a^*	3.26 ± 0.27 *^a^*	4.05 ± 0.09 *^b^*	32.42 ± 0.52 ^b^

Abbreviations: HWE: hot water extraction; SWE: subcritical water extraction; L: lightness; a: red/green coordinate; b: yellow/blue coordinate; C: chroma; H: hue. Values correspond to mean ± SD from three independent experiments. Different letters (a–d, *a*–*e*) denote a statistically significant difference (*p* < 0.05).

**Table 6:** Antioxidant activity of green seaweed hydrolysates.

Green Seaweed	Conditions	ABTS (AAE mg/g)	Total Antioxidant (TE mg/g)
*C. racemosa*	HWE	0.09 ± 0.05 ^d^	0.18 ± 0.03 ^d^
	SWE 110 °C	0.11 ± 0.03 ^d^	0.16 ± 0.04 ^d^
	SWE 150 °C	1.12 ± 0.05 ^c^	1.63 ± 0.07 ^c^
	SWE 190 °C	3.48 ± 0.10 ^b^	5.08 ± 0.09 ^b^
	SWE 230 °C	5.45 ± 0.11 ^a^	8.03 ± 0.06 ^a^
*U. lactuca*	HWE	0.15 ± 0.05 *^e^*	0.22 ± 0.07 *^d^*
	SWE 110 °C	0.22 ± 0.03 *^d^*	0.32 ± 0.05 *^d^*
	SWE 150 °C	0.40 ± 0.05 *c*	3.37 ± 0.07 *^c^*
	SWE 190 °C	7.09 ± 0.00 *^b^*	10.30 ± 0.00 *^b^*
	SWE 230 °C	8.14 ± 0.02 *^a^*	11.82 ± 0.02 *^a^*

Abbreviations: HWE: hot water extraction; SWE: subcritical water extraction; AAE: ascorbic acids equivalents; TE: trolox equivalents. Values correspond to mean ± SD from three independent experiments. Different letters (a–d, *a*–*e*) denote a statistically significant difference (*p* < 0.05).
